# Store-Operated Ca^2+^ Entry (SOCE) and Purinergic Receptor-Mediated Ca^2+^ Homeostasis in Murine bv2 Microglia Cells: Early Cellular Responses to ATP-Mediated Microglia Activation

**DOI:** 10.3389/fnmol.2016.00111

**Published:** 2016-10-28

**Authors:** Daniel F. Gilbert, Martin J. Stebbing, Katharina Kuenzel, Robyn M. Murphy, Evelyn Zacharewicz, Andreas Buttgereit, Leanne Stokes, David J. Adams, Oliver Friedrich

**Affiliations:** ^1^Department of Chemical and Biological Engineering, Institute of Medical Biotechnology, Friedrich-Alexander-Universität Erlangen-NürnbergErlangen, Germany; ^2^Erlangen Graduate School in Advanced Optical Technologies, Friedrich-Alexander-Universität Erlangen-NürnbergErlangen, Germany; ^3^Health Innovations Research Institute, Royal Melbourne Institute of Technology University, MelbourneVIC, Australia; ^4^Department of Biochemistry and Genetics, La Trobe Institute for Molecular Science, La Trobe University, MelbourneVIC, Australia

**Keywords:** BV2 microglia, calcium, store-operated calcium entry, ATP, multiphoton imaging, high-content screening, minocycline

## Abstract

Microglia activation is a neuroinflammatory response to parenchymal damage with release of intracellular metabolites, e.g., purines, and signaling molecules from damaged cells. Extracellular purines can elicit Ca^2+^-mediated microglia activation involving P2X/P2Y receptors with metabotropic (P2Y) and ionotropic (P2X) cell signaling in target cells. Such microglia activation results in increased phagocytic activity, activation of their inflammasome and release of cytokines to sustain neuroinflammatory (so-called M1/M2 polarization). ATP-induced activation of ionotropic P2X4 and P2X7 receptors differentially induces receptor-operated Ca^2+^ entry (ROCE). Although store-operated Ca^2+^ entry (SOCE) was identified to modulate ROCE in primary microglia, its existence and role in one of the most common murine microglia cell line, BV2, is unknown. To dissect SOCE from ROCE in BV2 cells, we applied high-resolution multiphoton Ca^2+^ imaging. After depleting internal Ca^2+^ stores, SOCE was clearly detectable. High ATP concentrations (1 mM) elicited sustained increases in intracellular [Ca^2+^]_i_ whereas lower concentrations (≤100 μM) also induced Ca^2+^ oscillations. These differential responses were assigned to P2X7 and P2X4 activation, respectively. Pharmacologically inhibiting P2Y and P2X responses did not affect SOCE, and in fact, P2Y-responses were barely detectable in BV2 cells. STIM1S content was significantly upregulated by 1 mM ATP. As P2X-mediated Ca^2+^ oscillations were rare events in single cells, we implemented a high-content screening approach that allows to record Ca^2+^ signal patterns from a large number of individual cells at lower optical resolution. Using automated classifier analysis, several drugs (minocycline, U73122, U73343, wortmannin, LY294002, AZ10606120) were tested on their profile to act on Ca^2+^ oscillations (P2X4) and sustained [Ca^2+^]_i_ increases. We demonstrate specific drug effects on purinergic Ca^2+^ pathways and provide new pharmacological insights into Ca^2+^ oscillations in BV2 cells. For example, minocycline inhibits both P2X7- and P2X4-mediated Ca^2+^-responses, and this may explain its anti-inflammatory action in neuroinflammatory disease. As a technical result, our novel automated bio-screening approach provides a biomedical engineering platform to allow high-content drug library screens to study neuro-inflammation *in vitro*.

## Introduction

Immune-modulatory microglia cells constitute about one fifth of the brain glia population and screen the brain parenchyma for tissue damage. During brain injury, purines (ATP, ADP) released by damaged brain cells activate microglia by stimulating purinergic receptors, either metabotropic G protein-coupled P2Y (Koizumi et al., 2007; De Simone et al., 2010) or ionotropic P2X receptors (James and Butt, 2002; Raouf et al., 2007). Microglia activation, motility towards injury sites and phagocytosis are mediated by calcium-dependent purinergic signaling, either via P2Y-induced Ca^2+^ release from internal stores or P2X-induced plasmalemmal Ca^2+^ entry (James and Butt, 2002; Ohsawa et al., 2007; Langfelder et al., 2015; Sunkaria et al., 2015). Fine-tuning of Ca^2+^-mediated cellular microglia activation, such as (i) during subtle ATP exposure seen in normal neuronal/astrocytes homeostasis, or (ii) during massive ATP exposure during excessive parenchymal cell damage, is predominantly reflected by the differential expression of P2X/Y receptor isoforms. For instance, sepsis can reverse the transcriptomic profile for P2X4 and P2X7 receptors, the major two P2X isoforms expressed on murine BV2 microglia cells (Raouf et al., 2007). Both ionotropic receptors contribute significantly to neuro-inflammation, with P2X4 isoforms having a higher affinity for extracellular ATP in the lower <100 μM range, whereas P2X7 isoforms predominantly respond to higher ATP concentrations in the mM range (Chessell et al., 1998; Raouf et al., 2007). Intriguingly, this ATP-sensitivity is also reflected by a differential Ca^2+^ entry response, with low ATP concentrations inducing a prompt and large but recoverable Ca^2+^ transient that does not recover after high ATP concentrations (Visentin et al., 1999). In particular, activating P2X7 receptors induces sustained formation of macropores and release of pro-inflammatory cytokines IL-1ß and TNF-α (James and Butt, 2002; Bartlett et al., 2014). While Ca^2+^ activation patterns (intracellular release, Ca^2+^ influx) are important in discriminating microglia responses via different purinergic receptors and signaling cascades, other sources of Ca^2+^ entry have also become of interest. In addition to receptor-operated Ca^2+^ entry (ROCE) in microglia cells, store-operated Ca^2+^ entry (SOCE) was also recently identified as an integral part of purinergic activation in primary mouse microglia (Heo et al., 2015; Michaelis et al., 2015). SOCE is an elementary Ca^2+^ regulatory mechanism that translates detection of internal store (endoplasmic reticulum, ER) Ca^2+^ contents to plasmalemmal Ca^2+^ entry via the sensory right-handed EF protein Stim (stromal interaction molecule), and a ‘Ca^2+^ release-activated Ca^2+^ channel’ (CRAC), called Orai, that is assembled on the sarcolemma when ER Ca^2+^ is low.

Ca^2+^ fluorescence recordings to study different Ca^2+^-dependent pathways in individual primary microglia cells can become exhaustive as exigent preparation and culturing usually limits their use in high-throughput/content assays. The murine BV2 cell line, an immortalized microglial cell line, is frequently used as a reliable alternative model system for primary microglia cultures, and it also reproduces complex cell–cell interaction studies (Henn et al., 2009). However, it is currently not known whether SOCE is present in BV2 cells as in primary murine microglia (Michaelis et al., 2015), and how their differential Ca^2+^ responses to extracellular purines compare to the latter. Thus, we pursued two objectives: (1) to provide evidence for the presence of SOCE and the differential pharmacokinetics of purinergic Ca^2+^ signaling in BV2 cells, and (2) to establish a high-content screening platform suitable for large-scale pathway screening in BV2 cells.

## Materials and Methods

### Pharmacological Reagents

Na_2_ATP, AZ106 (AZ10606120) and minocycline were from Tocris Bioscience (Bristol, UK). Thapsigargin, U73122, U73343, LY294 (LY294,002) and wortmannin were from Sigma–Aldrich (Castle Hill, NSW, Australia). Drugs were added to final solutions from frozen stock aliquots.

### BV2 Cell Culture

BV2 cells (American Type Culture Collection, ATCC; Manassas, VA, USA) were cultured (37°C, 5% CO_2_) in DMEM, supplemented with 10% fetal bovine serum and penicillin (100U/ml)/streptomycin (100 mg/ml) (Invitrogen) and passaged every 2–3 days (60–80% confluence). For high-content functional imaging, ∼5,000 BV2 cells were plated into each well of a 384 multi-well plate (BD Falcon) and incubated for 24 h. Wells typically contained 7.5–10 × 10^3^ cells at the time of experiments. For fluo-4 two-photon recordings, BV2-seeded round cover-slips were directly used for staining and imaging.

### Protein Biochemistry of Stim1S and Stim2

BV-2 cells were incubated in medium containing various treatments for 3 h before whole cell extracts were prepared in 1x SDS loading buffer. Denatured samples and calibration curves (4, 8, and 16 μl of mixed whole cell preparations) were loaded onto 26 well, 4–15% Criterion TGX Stain Free gels (Bio-Rad, Hercules, CA, USA) and run for 4 min at 200 V. Protein was transferred to nitrocellulose membrane at 100 V for 30 min, incubated in Pierce Miser solution (Pierce, Rockford, IL, USA) and blocked. Primary antibodies Stim1S (1:500, mouse monoclonal, #610954, BD Biosciences, Lexington, Kentucky, USA) and Stim2 (1:1000, rabbit polyclonal S8572, Sigma–Aldrich, Sydney, NSW, Australia) were diluted in 1% BSA-PBS with 0.025% Tween and membranes incubated overnight at 4°C and 2 h at RT. After incubating with a secondary antibody (goat anti-mouse IgG, goat anti-rabbit IgG, HRP conjugated, 1:20,000), the membrane was coated with chemiluminescent substrate (West Femto, Thermo Scientific, Rockford, IL, USA). Each gel-lane was normalized to the total protein of that sample, determined from Stain Free gel, then to the three point calibration curve thereby providing a quantitative approach to western blotting (Murphy and Lamb, 2013). Data from most samples are the average of duplicate runs.

### Fluo-4 Two-Photon Fluorescence Recordings

A Nikon A1 multiphoton microscope equipped with a Coherent^®^ Ti:Sa laser was used. BV2 cells were loaded with 5 μM fluo-4 AM (Invitrogen, Germany) in normal saline (in mM: NaCl 140, KCl 5, CaCl_2_ 2, MgCl_2_ 1, HEPES 10 and glucose 10, pH 7.4) for 20 min at 37°C and washed. Before recordings, cells were either flushed with Ca^2+^ free medium for 5 min or kept in normal saline, depending on the experiment. Two-photon excitation was done at 900 nm, and backscattered fluorescence recorded in de-scanned mode. Fluorescence intensity was recorded with a photomultiplier tube after passing through a 525/50 bandpass filter. Time lapse 512 pixels × 512 pixels recordings (XYT) were performed at 0.937 fps. Laser power was <3%. All settings were kept constant between recordings. Chemicals were added from stock solutions to the bath medium to obtain the final concentrations indicated. XYT image stacks were manually analyzed, assigning ROIs to individual BV2 cells and extracting temporal fluo-4 profiles. Individual traces were normalized to the average fluorescence within the first 10 s under initial resting conditions and converted to relative changes Δ*F*/*F*_0_ for group analysis. Individual data traces are mostly presented as raw fluorescence intensities.

### High-Content Functional Imaging

One and half hours before experiments, culture medium was removed from 384-well plates and replaced by normal saline (control solution), supplemented with 2 μM pluronic acid and 2 μM fluo 4-AM (Molecular Probes). After staining, solution was replaced by control solution. For receptor stimulation, control solution contained 1, 10, 100, or 1,000 μM final ATP concentration ([ATP]). For drug screening, control and agonist (ATP) solutions were supplemented with minocycline (1, 10, and 100 μM), AZ106 (10 μM) U73122 (5 μM), U73343 (5 μM), LY294002 (5 μM) or wortmannin (100 nM), respectively. Three hundred and eighty-four-well plates were placed onto the motorized stage of an ImageXpress^®^ Micro XLS System (Molecular Devices, USA) and cells were imaged with a 10× objective. A mercury arc lamp, passing through a GFP dichroic mirror, was used to excite fluo-4 fluorescence and imaged with a CCD camera. The image resolution after 2 × 2 binning was 696 pixels × 520 pixels. The experimental protocol involved manual addition of 10 μl agonist or 30 μl agonist/drug solution at a rate of ∼0.5 ml/min, then imaging of each well 380 times at a rate of 2 Hz (300 ms exposure time).

### Image Analysis from High-Content Screening

Fluorescence images of cells were segmented and quantitated using a modified version of DetecTIFF^®^ software (Gilbert et al., 2009). Briefly, an averaged image was calculated from all images recorded within a single well, segmented using an iterative size- and intensity-based thresholding algorithm, and then the fluorescence signal of identified cells was calculated as the mean of all pixel values within the cell area. To evaluate drug effects on the resting intracellular Ca^2+^ concentration, the fluorescence intensity of identified cells was calculated from the first image of the image series as the mean of all pixel values within the cell area, using the averaged image as mask for quantitative analysis. The drug effect was calculated as the arithmetic mean of the mean fluorescence intensity of all cells within the averaged image. Cell morphology was quantified as a function of elongation (*elongation factor*) as:

$$\begin{array}{c} \mathrm{Elongation}factor =\frac{\mathrm{Max}{\mathrm{intercept}}_{\mathrm{cell}}}{\mathrm{Mean}\mathrm{perpendicular}{\mathrm{intercept}}_{\mathrm{cell}}} \end{array}$$

An *elongation factor* of one represents a perfectly round cell, indicating a strong effect on cellular fitness or viability. A high value indicates normal morphology and unaffected viability. The drug effect was calculated as the arithmetic mean of the *elongation factors* of all cells. Individual [Ca^2+^]_i_ responses were reconstructed from image series of the same cells exposed to a defined ATP concentration or a combination of ATP and a drug. Each image typically contained 100–500 fluorescent cells for each tested well. For functional analysis of time-resolved [Ca^2+^]_i_ responses and subsequent classification and phenotyping of cell populations, a set of measures was taken from single cell-derived data. Responses were fitted with the LabView VI *Curve Fitting Express VI* using polynomial model type. The function finds the set of polynomial fit coefficients that best represents the input data. Each response was fitted twice with polynomial orders set to a value of 8 and 25. Responses fitted with polynomial order 8 were further evaluated using the LabView VI *Peak Detector VI* to identify a sustained calcium signal, indicating macro-pore formation, and activation of BV2 microglia cells. Responses fitted with polynomial order value 25 were analyzed for identification of ‘*intermediate* [Ca^2+^]_i_ *oscillations*’ occurring (i) between a transient [Ca^2+^]_i_ signal and sustained [Ca^2+^]_i_ increase, or (ii) sporadically during the entire span of the measurement using the residual of the fit. In addition, the fast-Fourier-transform (FFT) power spectrum of [Ca^2+^]_i_ responses was calculated for each cell using the LabView VI *FFT Power Spectrum and PSD VI*. Data obtained from curve fitting, peak and threshold detection and spectral analysis were used to calculate a total of 27 features (**Table 1**). Those data were used for classification and phenotyping of cell populations.

**Table 1:** [Ca^2+^]_i_ response phenotype descriptors.

Name	Description
1stSmlPk	Number of small peaks first 90 s of experiment (width, threshold: 3, 3)
2ndSmlPk	Number of small peaks last 90 s of experiment (width, threshold: 3, 3)
ampNormPS	Aplitude of normalized power spectrum
ampPS	Aplitude of power spectrum
dF	Δ*F* of (start to end of experiment)
dFP	Δ*F* (valley, peak) of large peak
dTP	Δ*t* (valley, peak) of large peak
expFitCoeff	Coefficients that describe the best polynomial fit
expFitErr	Mean squared error of the best fit (polyn., order 8)
gauCentrNormPS	Center of the fitted model (Gauss fit of norm. power spectrum)
gauCentrPS	Center of the fitted model (Gauss fit of power spectrum)
gauNormRes	Weighted mean error of the fitted model (Gauss fit of norm. power spectrum)
gauNormSD	Standard deviation of the fitted model (Gauss fit of norm. power spectrum)
gauRes	Weighted mean error of the fitted model (Gauss fit of power spectrum)
gauSD	Standard deviation of the fitted model (Gauss fit of power spectrum)
lgePk	Number of large peaks (width, threshold: 50, 50)
meanF	Mean value of fluorescence signal
meanNormPS	Mean value of normalized power spectrum
meanPS	Mean value of power spectrum
medF	Median value of fluorescence signal
medNormPS	Median value of norm. power spectrum
medPS	Median value of power spectrum
peakPS	Peak location of power spectrum
SDF	Standard deviation of fluorescence signal
SDNormPS	Standard deviation of norm. power spectrum
SDPS	Standard deviation of power spectrum
Variance	Variance of fluorescence signal

*Each row corresponds to an element of the descriptor vector.*

### Classifying of [Ca^2+^]_i_ Responses and Functional Phenotyping

To identify distinct functional phenotypes, a three-stage procedure was applied. First, a training set comprising a total of 1,709 single cell-derived [Ca^2+^]_i_ responses was manually assigned to one of three classes using a custom-written LabView-based software. The classes were named and characterized as follows:

• *Oscillation* & *Peak* (*n* = 333); response displays ‘intermediate [Ca^2+^]_i_ oscillations,’ followed by sustained [Ca^2+^]_i_ increase (**Figure 5A**, upper panel) indicating macro-pore formation and activation of BV2 microglia
• *Peak* (*n* = 536); response displays sustained [Ca^2+^]_i_ increase only (**Figure 5A**, middle panel) indicating macro-pore formation and activation of BV2 cells
• *Other* (*n* = 840); response displays none of the above listed characteristics, but any other type of [Ca^2+^]_i_ signal (**Figure 5A**, lower panel).

Next, a phenotype classification model was computed by a 10-fold, cross-validation of the training set using J48 decision tree algorithm available in WEKA 3.6 software (Hall et al., 2009). We accurately classified 1,683 or 98.5% of all responses correctly. A confusion matrix is shown in **Table 2**. Finally, all single cell-based ATP-induced [Ca^2+^]_i_ responses recorded in the presence of different ATP concentrations and ATP/drug combinations were analyzed based on the computed classification model.

**Table 2:** Result of a 10-fold cross-validation of training sets using a J48 decision tree algorithm for calculating a model for phenotyping of ATP-induced [Ca^2+^]_i_ responses in BV2 cells.

	Oscillation & Peak	Peak	Other
Oscillation & Peak	331	0	2
Peak	0	523	9
Other	2	13	829
Acc. %	99.4	97.6	98.7

*Columns/rows: real/predicted classes.*

### Statistical Data Analysis

Data were processed using MS Office 2010, SigmaPlot, Origin 7G, ImageJ and IrfanView. Biochemical protein data was analyzed using Prism GraphPad. Statistical analysis was done based on one-way and two-way ANOVA tests, checking for data normality and performing *post hoc* tests (Dunn or Bonferroni method). Asterisks in the graphs indicate significance levels; ^∗^*p* ≤ 0.05, ^∗∗^*p* ≤ 0.01, ^∗∗∗^*p* ≤ 0.001.

## Results

### Store-Operated and ATP-Mediated Ca^2+^ Entry in Murine BV2 Cells

**FIGURE 1:**
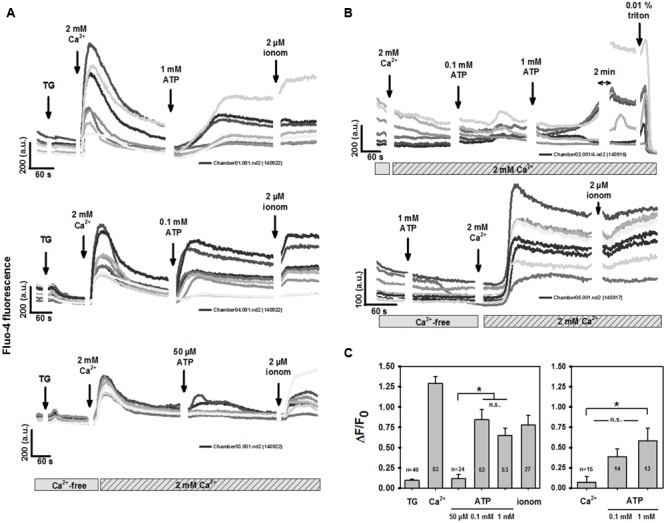
**Murine BV2 microglia cells show robust store-operated Ca^2+^ entry (SOCE). (A)** Representative recordings of two-photon excited fluo-4 Ca^2+^ fluorescence from several individual BV2 cells. Under Ca^2+^-free conditions, thapsigargin (TG) was introduced to empty internal stores, followed by addition of 2 mM external Ca^2+^ which induced a robust Ca^2+^-entry with transient rise in intracellular global Ca^2+^. Following decline of SOCE, extracellular addition of ATP induced a delayed sustained rise in Ca^2+^ in a concentration-dependent manner. This was almost complete for 0.1 mM and 1 mM ATP as judged from the response towards Ca^2+^ permeabilization by ionomycin **(C)**. **(B)** Omitting store-depletion prior to introducing external Ca^2+^ did not induce any SOCE response. External addition of ATP induced Ca^2+^ influx into BV2 cells because addition of ATP under Ca^2+^-free conditions (lower panel) abrogated the rise in Ca^2+^ fluorescence until extracellular Ca^2+^ was added. **(C)** Statistical analysis of SOCE and ATP-mediated Ca^2+^ entry. The right panel refers to experiments such as shown in **(B)**, upper panel, where Ca^2+^ was added externally to BV2 cells without prior store-depletion.

To distinguish purinergic receptor-mediated Ca^2+^ entry from intracellular ER Ca^2+^ release, BV2 cells were incubated in Ca^2+^-free media and internal stores emptied by 5 μM thapsigargin (TG), while two-photon excited fluo-4 fluorescence was recorded. Two-photon microscopy has the advantage of an excitation volume in the μl^3^ range, which substantially reduces dye bleaching and allows cellular long-term fluo-4 recordings. **Figure 1A** shows recordings from three different BV2 dishes. There was a small transient increase in fluo-4 fluorescence after TG application. Adding 2 mM external Ca^2+^ elicited a robust Ca^2+^ transient, while Ca^2+^ stores remained emptied. This is a hallmark of store-operated Ca^2+^ entry (SOCE). Applying ATP after an almost complete decline of the SOCE transient evoked a sustained increase in intracellular fluo-4 fluorescence in a concentration-dependent manner. This was mediated by purinergic receptor-operated Ca^2+^ entry (ROCE) to almost maximum Ca^2+^ permeability for ATP concentrations starting from 0.1 mM, as determined from subsequent Ca^2+^ ionophoresis (2 μM ionomycin). We confirmed this in several dishes in more than 20 cells for each condition (**Figure 1C**). Lower ATP concentrations (50 μM) evoked significantly smaller peak Δ*F*/*F*_0_ levels than concentrations of 0.1 and 1 mM ATP, for which levels were similar to those in ionomycin-induced results. Not emptying internal stores and adding external Ca^2+^ again did not induce SOCE (**Figure 1B**), but did reproduce the behavior of purinergic ROCE with empty stores, albeit at a somewhat lower level (**Figures 1B,C**). One explanation could be that an ongoing blockade of SERCA with TG resulted in a small residual component of SOCE to the resulting Ca^2+^ entry (mainly ROCE). **Figure 1B** shows that also in the case of sole purinergic ROCE activation (without prior store depletion), the sustained intracellular Ca^2+^ increase was almost complete. This is because the subsequent unspecific breakdown of the sarcolemma by triton X-100 evoked only a brief minor increase before all of the dye dissipated from the cells, resulting in a loss of fluorescence. In other experiments, we addressed the question of whether purinergic receptor activation without external Ca^2+^, but with internal stores not emptied, would induce PLC-mediated (P2Y) signaling to empty internal stores and transiently increase fluo-4 fluorescence. **Figure 1B** shows a representative Ca^2+^ response demonstrating that this did not occur. Purinergic ROCE was only detected once external Ca^2+^ was re-introduced. Again, ATP-induced Ca^2+^ entry was almost complete compared with subsequent Ca^2+^ ionophoresis.

**FIGURE 2:**
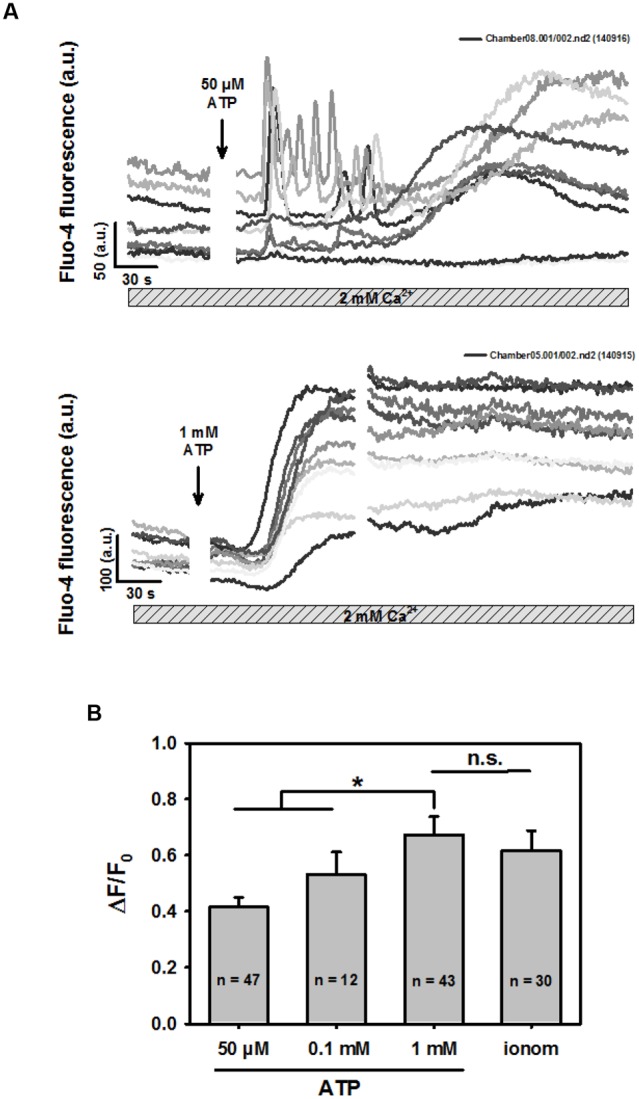
**ATP concentration-response for sustained rise in intracellular Ca^2+^ in BV2 cells bathed in 2 mM external Ca^2+^. (A)** Examples of two-photon fluo-4 recordings in several BV2 cells and P2X receptors stimulated with low (50 μM) and high concentrations (1 mM) of ATP. In particular with low ATP concentrations, Ca^2+^ oscillations were occasionally observed, followed by a sustained rise in Ca^2+^. For larger ATP concentrations, almost exclusively sustained Ca^2+^ responses were observed in this setting. **(B)** Statistical analysis of ATP-induced ROCE. Low ATP concentration-induced Ca^2+^-peak of the sustained response was significantly smaller compared with the response to 1 mM ATP. The latter induced a response as maximal as by Ca^2+^ ionophoresis with 2 μM ionomycin.

When we further differentiated early purinergic ROCE responses in BV2 cells in highly spatio-temporally resolved multiphoton recordings, two distinct patterns were observed: oscillatory Ca^2+^ fluctuations and the sustained slow Ca^2+^ increase. Both patterns were immediately initiated after ATP addition. **Figure 2A** shows a representative immediate oscillatory pattern followed by the sustained increase in intracellular Ca^2+^ due to purinergic ROCE (stores not emptied) over several minutes. Such oscillation patterns were rarely observed in response to 0.1 mM ATP in multiphoton experiments. They were sometimes observed with 50 μM ATP, still at a low frequency (∼15% of experiments). **Figure 2B** shows the concentration-response relationship for the three ATP concentrations tested in the scenario of recording purinergic ROCE with untouched internal Ca^2+^ stores, and in the presence of 2 mM Ca^2+^ (BV2 cells never exposed to Ca^2+^-free environment in experiments of **Figure 2**). Δ*F*/*F*_0_ was significantly larger at 1 mM ATP than at lower concentrations. Due to rare detection in highly resolved two-photon experiments, we further studied Ca^2+^ oscillations in a setting of low-resolution, high-content screening (see below).

### Minocycline Inhibits High ATP-Mediated Purinergic ROCE Response in BV2 Cells

**FIGURE 3:**
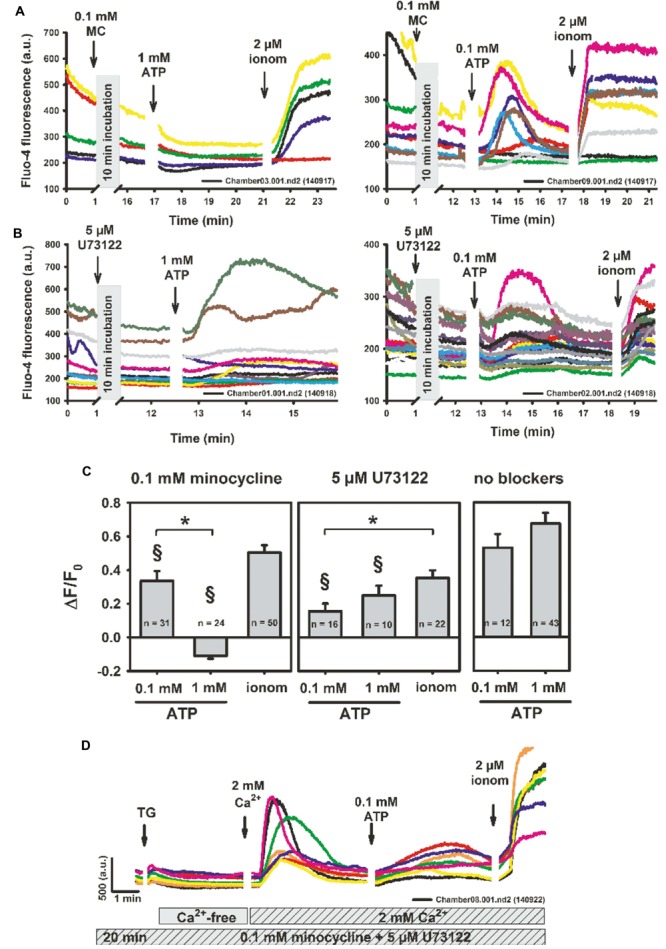
**Minocycline specifically abrogates P2X-mediated purinergic response in BV2 cells (P2X7 > P2X4) but does not affect store-operated Ca^2+^ entry. (A)** Representative recordings showing two-photon fluo-4 Ca^2+^ fluorescence recordings in BV2 cells after 10 min of incubation with 0.1 mM minocycline, followed by differential P2X activation with ATP. Minocycline itself does not induce a Ca^2+^ response. Only low/intermediate concentrations of ATP (0.1 mM) induce extracellular Ca^2+^ influx whereas the 1 mM response is completely blunted. However, statistical analysis already shows a reduction of low ATP-responses by minocycline versus controls without blocker (^§^ *P* < 0.05) **(C)**. In contrast, neither low nor high concentrations of ATP are affected in their relative Ca^2+^ response by incubation with the PLC inhibitor U73122, although Ca^2+^ responses were already significantly smaller as compared to controls **(B,C)**. **(D)** Representative recordings of two-photon fluo-4 fluorescence after 20 min of pre-incubation with 0.1 mM minocycline and 5 μM U73122 followed by store depletion with thapsigargin, re-addition of 2 mM external Ca^2+^ and P2X-activation with ATP. Neither U73122 nor minocycline inhibited SOCE in BV2 cells. ^∗^*P* < 0.05, as indicated. ^§^ *P* < 0.05 vs. control (‘no blockers’).

The broad-spectrum tetracycline antibiotic, minocycline, is a known microglia activation inhibitor (Wang W. et al., 2015), with inhibitory action on P2X4 and P2X7 receptors (Stebbing, unpublished observations). To further explore its mechanisms of action on purinergic receptor activation and Ca^2+^ homeostasis, we performed two-photon Ca^2+^ fluorescence experiments in the presence of 0.1 mM minocycline and varying ATP concentrations. Pre-incubating BV2 cells with minocycline completely abolished the high [ATP]-induced response (1 mM), but not the ROCE oscillatory responses to lower concentrations (0.1 mM) (**Figure 3A**). However, even at the lower concentration, ATP-evoked ROCE Δ*F*/*F*_0_ levels were already significantly reduced compared to the Ca^2+^ responses seen at 0.1 mM ATP under control conditions (no inhibitors present) and also remained well below the levels observed after Ca^2+^ permeabilisation with ionomycin (**Figures 3A,C**). Minocycline alone did not induce any Ca^2+^ responses (**Figure 3A**). To address potential PLC pathway activation, we tested the PLC inhibitor U73122. The Ca^2+^ response in BV2 cells to either low or high ATP concentrations were also inhibited by incubation with 5 μM U73122 (U73343 not tested here) as compared to controls without inhibitor present, but were not significantly different under low- or high-ATP conditions (**Figures 3B,C**; all experiments with 2 mM external Ca^2+^). To test whether minocycline or U73122 affected SOCE, we conducted similar experiments to those outlined in **Figure 1**. As shown in a representative recording in several BV2 cells, neither minocycline nor U73122 blocked SOCE in BV2 cells (**Figure 3D**).

### Induction of Proteins of the SOCE Machinery by Purinergic Microglia Activation

**FIGURE 4:**
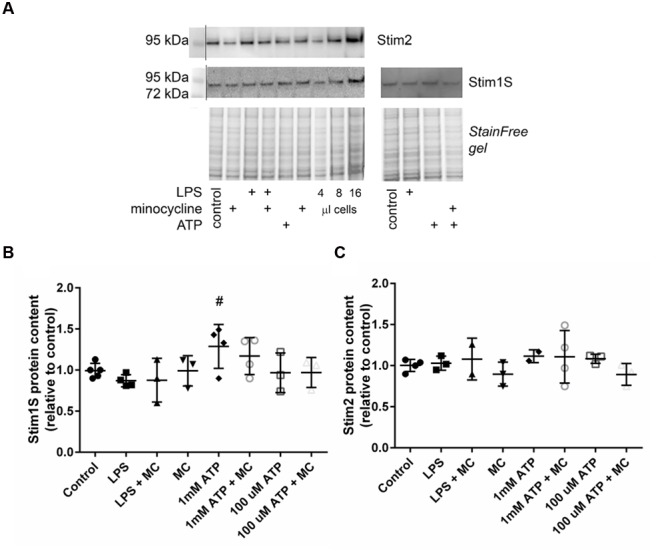
**Microglia activation by high ATP concentration (1 mM), but not LPS or low ATP, upregulates Stim 1S protein content. (A)** Representative Western blot probed for Stim 2 and Stim 1S with the corresponding stain-free gel under various conditions of microglia activation (LPS, 100 μM ATP, 1 mM ATP) and pre-treatment with minocycline. Group data analyses reveal significant upregulation of Stim 1S that is blunted by minocycline for high [ATP] (1 mM) treatment only (^#^ indicates the significance level, *p* = 0.0352 using a one way ANOVA) **(B)**, whereas no effect was observed for any treatment on Stim 2 protein content **(C)**.

To verify the effect of microglia activation on recruitment of SOCE at the protein level, quantitative Western blot analyses were carried out on BV2 cell lysates after differential treatment with microglia activators (LPS, ATP) and M1 polarization (minocycline) inhibitors. **Figure 4A** shows a representative gel and corresponding blots probed for Stim1S and Stim2: both are present in control BV2 cells, and 1 mM ATP stimulation appears to increase the amount of Stim1S. Compared to controls, only 1 mM ATP significantly upregulated Stim 1S protein content, and only minor or no changes were observed with 0.1 mM ATP or LPS, respectively (**Figure 4B**). Also, pre-treatment with minocycline blunted the upregulation of Stim1S. None of the microglia activation treatments (LPS, ATP) produced any upregulation in protein content for Stim 2 (**Figure 4C**).

### High Content Ca^2+^ Fluorescence Assay for Extended Compound Screening of Oscillatory and Sustained Early Purinergic Ca^2+^ Responses (ROCE) in BV2 Cells

Although the above shown two-photon excitation approach provides a high-resolution metrology to study Ca^2+^ homeostasis in single BV2 cells, in particular less frequent event patterns, like Ca^2+^ oscillations seen during ATP BV2 activation, are difficult to statistically evaluate. To address this and to enable a high-content screening for Ca^2+^ responses, we established an assay for single cell-based, high-content, functional screening of ATP-induced [Ca^2+^]_i_ responses.

### Functional Phenotyping of ATP-Induced [Ca^2+^]_i_ Responses in BV2 Cells

**FIGURE 5:**
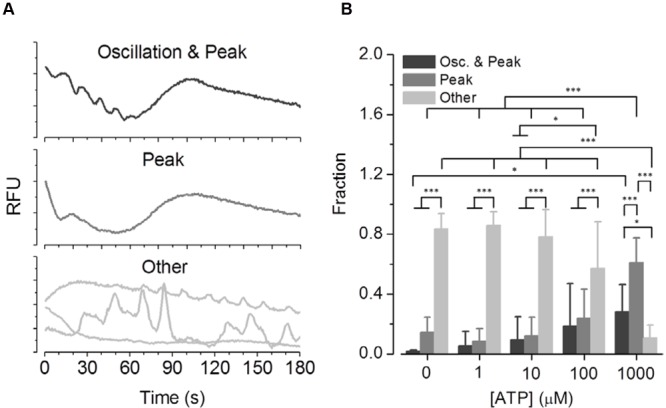
**Phenotypic analysis of ATP concentration-dependent functional classes in BV2 cells. (A)** Representative [Ca^2+^]_i_ responses of the three classes *Oscillation* & *Peak, Peak* and *Other* measured in BV2 cells in a high-content setting. **(B)** Fraction of cells exposed to 0 (*N* = 3 dishes), 1 (*N* = 10) 10 (*N* = 10), 100 (*N* = 11), and 1,000 μM ATP (N = 12), assigned to one of the three classes. The average fraction of cells assigned to the class *Other* decreases with increasing ATP concentrations, whereas the average fractions of cells assigned to the classes *Oscillation* & *Peak* and *Peak* increase with increasing ATP concentrations.

To evaluate the fraction and concentration-dependence of ATP-induced ‘intermediate [Ca^2+^]_i_ oscillations’, BV2 cells were prepared for screening experiments in 384-well plates. **Figure 5A** shows a selection of representative [Ca^2+^]_i_ responses of the three classes *Oscillation* & *Peak* (upper panel), *Peak* (middle panel), and *Other* (lower panel). **Figure 5B** shows the fraction statistics of BV2 cells exposed to 0 (*n* = 3 replica), 1 (*n* = 10), 10 (*n* = 10), 100 (*n* = 11), and 1,000 μM ATP (*n* = 12), assigned to one of the three classes. The average fraction of cells assigned to *Other* decreases with increasing ATP concentration, whereas the average fractions of cells assigned to *Oscillation* & *Peak* and *Peak* increase with increasing ATP concentration. These data clearly demonstrate that the established screening assay enables parallel recordings of ATP-induced [Ca^2+^]_i_ responses in many BV2 cells and functional phenotyping and quantification of the fraction of cells displaying ‘intermediate oscillations.’ This high-content assay is thus, more robust in quantifying low probability *intermediate oscillations* as compared to the high-resolution but low-content, two-photon experiments when recording from less cells.

### Phenotypic Profiling of ATP-Induced [Ca^2+^]_i_ Responses in BV2 Cells Exposed to Various Drugs

**FIGURE 6:**
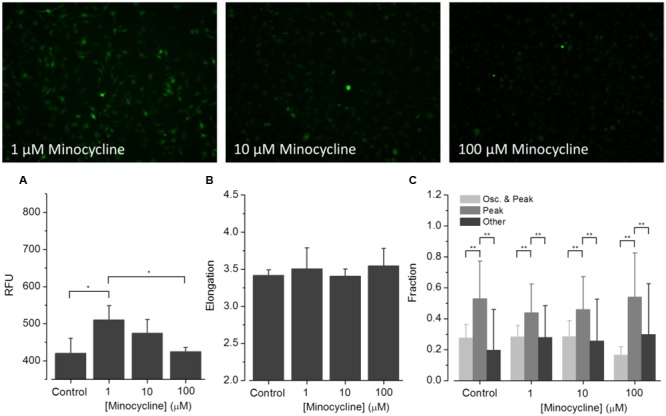
**Effect of minocycline on ATP-dependent Ca^2+^ signaling.** Fluorometric **(A)** and morphometric **(B)** analysis as well as functional phenotyping **(C)** of ATP-induced [Ca^2+^]_i_ responses in minocycline-exposed BV2 cells. The relative fluorescence intensity (RFU) depicted in the histogram in **(A)** represents the fluorescence intensity at experiment initiation, i.e., under control conditions, or in presence of small molecular drugs, and was analyzed at the level of single cells and subsequently averaged for individual images. The fluorometric analysis indicates declining ATP-induced [Ca^2+^]_i_ responses from low to high minocycline concentrations. Morphological analysis, i.e., quantification of the cells’ elongation as an indicator of cellular viability revealed no difference of drug treated compared to control cells **(B)**. ATP-induced [Ca^2+^]_i_ responses were not perturbed by any of the used minocycline concentrations **(C)**.

**FIGURE 7:**
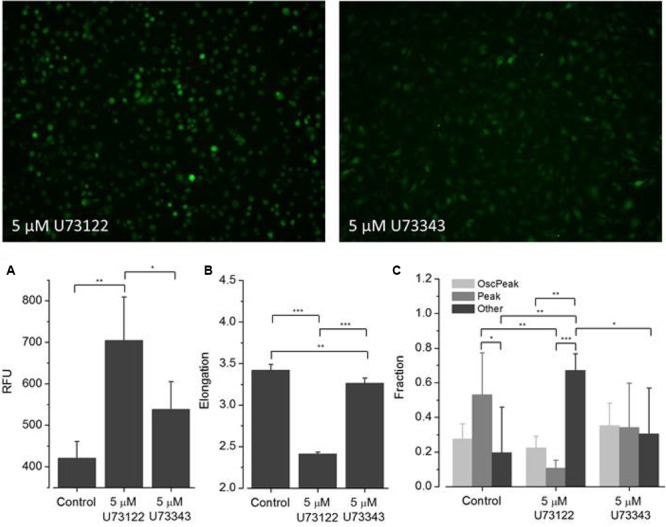
**Effect of U73122 and U73343 on ATP-dependent Ca^2+^ signaling.** Fluorometric **(A)** and morphometric **(B)** analysis and functional phenotyping **(C)** of ATP-induced [Ca^2+^]_i_ responses in U73122- and U73343-exposed BV2 cells indicating perturbed Ca^2+^ signaling upon drug treatment. In cells exposed to U73122 (5 μM), the classes *Peak* and *Other* are significantly altered compared to controls whereas the fraction of cells assigned to the class *Oscillation* & *Peak* is not affected. [Ca^2+^]_i_ responses in U73343-exposed BV2 cells are not significantly altered compared to controls **(C)**.

**FIGURE 8:**
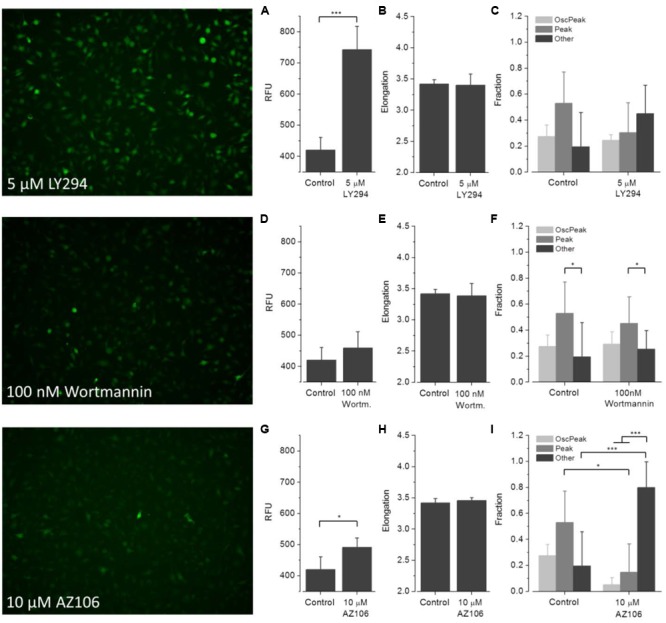
**Effect of LY294, wortmannin and AZ106 on ATP-dependent Ca^2+^ signaling.** Fluorometric **(A,D,G)** and morphometric **(B,E,H)** analysis and functional phenotyping **(C,F,I)** of ATP-induced [Ca^2+^]_i_ responses in drug exposed BV2 cells indicate perturbed Ca^2+^ signaling upon treatment with LY294 (5 μM) and AZ106 (10 μM), but not wortmannin (100 nM).

To assess the applicability of the established screening assay for employing ATP-induced ‘intermediate [Ca^2+^]_i_ oscillations’ as a readout for microglia activation-targeted compound screening, BV2 cells were incubated with one of six selected drugs (minocycline, U73122, U73343, wortmannin, LY294, AZ106) for at least 30 min. [Ca^2+^]_i_ responses were recorded after application of the agonist ATP (1 mM). **Figures 6**–**8** are representative images of BV2 cells shortly (∼10 s) after ATP application. They illustrate the effect of drug pre-incubation on Fluo-4 intensity and elongation of cells exposed to control solution. Respective drug concentrations are also shown in the bar graphs. Recorded time-resolved fluo-4 signals were automatically assigned to one *Oscillation* & *Peak, Peak* or *Other* classes and phenotype fractions were calculated from four independent experiments each. Averaged values (±SD) as shown in **Figures 6–8** are listed in **Table 3**.

**Table 3:** Average values (±SD) shown in **Figures 6**–**8**.

Drug	RFU	Elongation	Oscillation and Peak	Peak	Other
Control	420 ± 40	3.41 ± 0.07	0.27 ± 0.08	0.52 ± 0.24	0.19 ± 0.26
Minocycl. (1 μM)	510 ± 37	3.50 ± 0.28	0.28 ± 0.07	0.43 ± 0.18	0.27 ± 0.20
Minocycl. (10 μM)	474 ± 36	3.40 ± 0.09	0.28 ± 0.10	0.45 ± 0.21	0.25 ± 0.27
Minocycl. (100 μM)	424 ± 11	3.54 ± 0.23	0.16 ± 0.05	0.53 ± 0.28	0.29 ± 0.32
U73122	704 ± 104	2.41 ± 0.01	0.22 ± 0.06	0.10 ± 0.04	0.67 ± 0.09
U73343	538 ± 66	3.26 ± 0.06	0.35 ± 0.12	0.34 ± 0.25	0.30 ± 0.26
LY294	742 ± 75	3.39 ± 0.18	0.24 ± 0.04	0.30 ± 0.23	0.45 ± 0.21
Wortmannin	459 ± 52	3.38 ± 0.19	0.29 ± 0.09	0.45 ± 0.20	0.25 ± 0.14
AZ106	491 ± 30	3.45 ± 0.04	0.05 ± 0.05	0.14 ± 0.21	0.79 ± 0.19

(1) **Minocycline (**Figure 6**).** ATP-induced fluorescence intensity is increased at pre-incubation concentrations of 1 and 10 μM minocycline, compared to controls, and decreases with increasing drug concentration. The morphology of drug-exposed cells is comparable with control cells, indicating no effect on cellular viability. The fraction of *Oscillation* & *Peak* is diminished at a concentration of 100 μM minocycline compared to control and cells exposed to 1 and 10 μM minocycline. The fraction of *Peak* is reduced at concentrations of 1 and 10 μM minocycline but comparable with that of controls at 100 μM minocycline. The fraction of cells assigned to *Other* increased in drug- treated cells compared to controls.
(2) **U73122 and U73343 (**Figure 7**).** ATP-induced fluorescence intensity increased at a pre-incubation concentration to 5 μM U73122 or U73343 compared with control. The elongation of cells exposed to U73122 is markedly reduced, indicating round morphology and a pronounced effect on cellular viability. The fraction of *Oscillation* & *Peak* is reduced in cells treated with U73122 and is increased in cells exposed to U73343 compared to controls (U73343 commonly used as negative control for PLC inhibition). The fractions of *Peak* and *Other* are both reduced in cells exposed to U73122 or U73343.
(3) **Wortmannin (**Figure 8**).** ATP-induced fluorescence intensity is slightly increased at a pre-incubation concentration of 100 nM wortmannin compared to controls. The morphology and fractions of the three morphological classes of drug-exposed cells was comparable to controls, indicating no effect on cellular viability.
(4) **LY294 (**Figure 8**).** ATP-induced fluorescence intensity is markedly increased at a pre-incubation concentration of 5 μM LY294 compared to controls. The morphology of drug-exposed cells is comparable to controls, indicating no effect on cellular viability. The fractions of cells assigned to *Oscillation* & *Peak* and *Peak* are decreased, the fraction of *Other* is increased compared to controls.
(5) **AZ106 (**Figure 8**).** ATP-induced fluorescence intensity slightly increased at a pre-incubation concentration of 10 μM AZ106 compared to controls. The morphology of drug-exposed cells is similar to controls, indicating no effect on cellular viability. The fractions of cells assigned to *Oscillation* & *Peak* and *Peak* classes decreased but the fraction of *Other* increased compared to controls.

## Discussion

Purinergic activation of microglia in brain inflammatory, degenerative or tumor disease involves sensing the extracellular environment for excess ATP and a concentration-dependent activation of either G protein-coupled receptors (GPCRs) or direct activation of ion channels (James and Butt, 2002; Ohsawa et al., 2007; De Simone et al., 2010). Both processes increase intracellular Ca^2+^ levels, either through PLC-mediated release from internal stores (P2Y response) or increasing Ca^2+^ permeability of the sarcolemma (P2X response). The characteristics of Ca^2+^ fluctuations in activated microglia also depends on the differential activation of P2X4 and P2X7 receptors, the two major isoforms in these cells (Raouf et al., 2007; Bernier et al., 2012). Sustained activation of both isoforms leads to formation of membrane pores (macropore formation for P2X7; cf. Li et al., 2015). In the case of P2X4 activation, this does not require pannexin hemi-channel formation, so it is non-lethal to microglia cells (Bernier et al., 2012). Macropore formation is more likely to induce excessive microglia activation and even necrosis due to a large influx of organic molecules that sustain neuro-inflammation (Chessell et al., 1997; Takenouchi et al., 2005). This is documented by the strong P2X7-induced activation of microglia’s inflammasome, leading to sustained cytokine release (Ikeda et al., 2013). Although store-operated Ca^2+^ channels have been described in murine microglial MG5 cell lines and primary microglia, affecting the Ca^2+^ spiking activity in ATP-activated cells (Ikeda et al., 2013), the fine-tuning of purinergic signaling in primary microglia cells was only recently characterized (Michaelis et al., 2015). However, evidence for SOCE in BV2 cells, one of the most commonly used murine immortalized model cells for microglia, is still elusive, as was its interaction with ROCE.

### SOCE and ROCE in BV2 Microglial Cells

Our results demonstrate a large transient Ca^2+^ entry in BV2 cells after store-depletion. The amplitude of this SOCE was approximately ten times larger than the increase in Ca^2+^ fluorescence during store depletion with thapsigargin, which in turn is about two times larger than the relative increase after store depletion by CPA in primary microglia (Michaelis et al., 2015). Purinergic ROCE followed a clear ATP concentration-dependence with a marginal sustained response to 50 μM ATP, and a more complete sustained response to 1 mM ATP. The former is indicative of P2X4 activation, the latter of P2X7 activation (James and Butt, 2001). Not activating SOCE (without TG), also did not evoke Ca^2+^ entry when Ca^2+^ was added to a Ca^2+^-free solution. Interestingly, in this situation, purinergic ROCE responses were dampened compared with purinergic responses after SOCE (**Figure 1C**). This indicates an interaction between both processes. Alternatively, it may also indicate some residual contaminating contribution of SOCE to the global Ca^2+^ entry carried by ROCE after ATP application. However, such a contamination may be minor at best, as SOCE transients had already mostly declined before activation of ROCE (**Figure 1A**).

In primary microglia cells from Stim1/2 wt (^+/+^) and knockout (^-/-^) mice, where ablation of SOCE was also substantially reduced, a similar situation as in our BV2 cells was seen, with a sustained late ATP-induced ROCE response, most probably mediated by P2X, whereas P2Y receptor activation mediated an early transient Ca^2+^ spike, also present in Ca^2+^ free solution (Michaelis et al., 2015; their **Figure 4**). However, the latter represents a striking difference to the ATP-responses seen in our BV2 cells under Ca^2+^-free conditions, where ATP did not evoke any increase in intracellular Ca^2+^, indicative of blunted P2Y response in BV2 cells (**Figure 1B**). Although P2Y receptor expression has been recently confirmed (P2Y6 isoform; Zhu et al., 2015), we are not aware of any study that explicitly tested for Ca^2+^ liberation from the endoplasmic reticulum upon ATP-activated, P2Y-mediated PLC-pathways in BV2 cells. Although under our external 2 mM Ca^2+^ containing single cell experiments, where inhibition of PLC by U73122 did not have a significant effect on Ca^2+^ levels comparing low (0.1 mM) or high ATP (1 mM), it already significantly reduced those levels compared with controls (**Figure 3C**). While this may point to some involvement of P2Y-response to ATP, the results of missing Ca^2+^ responses in Ca^2+^-free external solution (**Figure 1B**) provides a stronger argument against a major involvement of those receptors in purinergic responses in BV2 cells. In that regard, BV2 microglia cells might be particularly suitable to study P2X-mediated purinergic Ca^2+^ responses and to elucidate putative cross-talks between SOCE and ROCE. Such a cross-talk is also compatible with P2X7 activation by 1 mM ATP (but not P2X4 or LPS activation) significantly upregulating Stim1S protein content, without changing Stim2 (**Figure 4**). Moreover, a fast translational control over Stim1 protein expression within ∼6 h treatment of BV2 cells with 1 mM ATP, sufficient to initiate protein upregulation, is apparent from our results. These findings suggest that upregulation of Stim1 protein abundance may be an additional regulatory mechanism of SOCE, which primarily involves the translocation of Stim1 along the ER membrane to juxtapose Orai channels to trigger SOCE. Treatment with LPS for such periods is obviously not enough to induce Stim overexpression which seems different to primary microglia treated with LPS for periods of 3–24 h, resulting in a ∼2.5-fold overexpression of Stim1 (Heo et al., 2015). An interesting similarity between our ROCE responses in BV2 cells and primary microglia cells (Michaelis et al., 2015) is reflected by their averaged Ca^2+^ trace profiles (from up to 30 cells) suggesting a more sustained ATP-mediated P2X Ca^2+^ response in Stim1^+/+^ primary microglia, while this seemed more transient for Stim2^+/+^ primary microglia (Michaelis et al., 2015; their **Figures 2B,C**, respectively) when stimulated with a fixed ATP concentration of 0.3 mM. Although not systematically investigated by them, one may speculate that the ROCE of sustained type associated with high ATP concentrations (from 0.1 mM) in our study may be related to interaction with Stim1 while the more transient and even oscillatory ATP-ROCE responses at lower ATP (<0.1 mM) may be regulated by Stim2. Thus, P2X4 may interact with the Stim2 machinery while P2X7 may do so with Stim1. Future studies using isoform-specific knockdown of Stim in BV2 are required to address this hypothesis. Nevertheless, using 0.1 mM ATP to stimulate ROCE, Heo et al. (2015) also observed sustained Ca^2+^ responses in their murine primary microglia model (Heo et al., 2015).

The broad-spectrum antibiotic minocycline inhibits microglia activation during brain inflammatory processes (Wang N. et al., 2015; Henry et al., 2008). It also blocks LPS-stimulated pro-inflammatory cytokine secretion in BV2 cells and reduces microglial toll-like-2 receptor expression (Henry et al., 2008). One potential mechanism of minocycline preventing pro-inflammatory microglial M1 polarization is through inhibited NF-κB upregulation (Kobayashi et al., 2013). Because purines released from inflamed or damaged parenchyma sustain M1 polarization via purinergic activation, its link between microglia inflammasome activation and Ca^2+^ signaling appears obvious. However, to date, this action of minocycline remained undetermined. Therefore, our results provide an important element to understand the actions of minocycline in microglia. We suggest minocycline prevents M1 polarization by blocking P2X4- and P2X7-mediated ROCE, which inhibits Ca^2+^-induced activation of pro-inflammatory pathways and the inflammasome (Stebbing, unpublished observations). From our study, minocycline appears to have higher blocking efficiencies for purinergic Ca^2+^ influx via P2X7 over P2X4 receptors (**Figure 3C**).

### Classification of ROCE Responses in BV2 Cells and a Readout-Platform for High-Throughput/Content Testing

In Ca^2+^-containing external solutions in which SOCE is not activated, purinergic ROCE was ATP concentration-dependent and maximal for high concentrations (≥1 mM), activating P2X7 receptors (**Figure 2B**). Selective Ca^2+^ permeabilization of the membrane confirmed no additional increase in fluo-4 fluorescence. In particular, for lower ATP concentrations, early Ca^2+^ oscillations occasionally occurred before the delayed sustained rise in Ca^2+^ fluorescence. These oscillations were rarely observed in our high-resolution two-photon imaging at high ATP concentrations, which suggests an association with P2X4 (but not P2X7) activation. Ca^2+^ oscillations have been reported to occur spontaneously in BV2 cells as a result of cell-cell communication mediated by extracellular messengers, i.e., ATP (Wu et al., 2013). ATP-evoked Ca^2+^ oscillations are not well characterized in microglia. In a recent study in microglia cells from a murine Alzheimer’s disease model, ATP induced a more transient response, whereas spontaneous signals co-occurred with Ca^2+^ oscillations in adjacent neurons and astrocytes (Brawek et al., 2014). As differential Ca^2+^ responses in microglia may be of paramount importance for differentially regulating purinergic response patterns, we sought to establish a classification system of ROCE-responses in a high-content setting. This has the advantage of more reliably following rather rare events that may be missed in single cell multiphoton recordings, because the latter focus on high spatial resolution in individual cells rather than on observations of large numbers of cells. During our multiphoton recordings, it already became apparent that there were at least two distinct Ca^2+^ responses (classes): *Oscillations & Peak* and *Peak*. We expanded the classes to include a third, *Other*, which summarized all other events. The assay has been applied here in a *proof-of-concept* study including ATP concentration-response experiments and functional imaging in cells exposed to a selection of drugs selectively perturbing ATP-dependent Ca^2+^ signaling. Our results revealed that the fraction of cells assigned to one of the three functional classes changed with increasing ATP concentrations, demonstrating that the experimental and analytic approaches for studying distinct ATP-dependent Ca^2+^ signaling in BV2 cells were applicable. The effects of minocycline, U73122, U73343, wortmannin, LY294 and AZ106 were subsequently tested on the Ca^2+^ responses.

Minocycline was reported to inhibit microglia activation by blocking NF-κB nuclear translocation (Giuliani et al., 2005). Our data from both the two-photon experiments and high-content studies indicate it directly inhibits early P2X-induced purinergic ROCE in BV2 cells. The observation of reduced and increased fractions of the *Oscillation* & *Peak* and *Peak* classes, respectively, at a concentration of 100 μM minocycline in large numbers of BV2 cells suggests the drug inhibits P2X4 receptors (Stebbing, unpublished observations). These results also indicate that oscillatory and sustained Ca^2+^ signals cannot be unambiguously attributed to either P2X4 or P2X7 receptors (**Figure 6**). Such assignment to either channel requires future work involving specific P2X-receptor knockout models or transient siRNA knock-down in expression systems.

U73122 affects ATP-induced Ca^2+^ signaling by inhibiting the transient [Ca^2+^]_i_ increase which involves PLC to release Ca^2+^ from intracellular stores (Takenouchi et al., 2005). Furthermore, U73122 inhibits P2X7-mediated sustained [Ca^2+^]_i_ increase, i.e., those [Ca^2+^]_i_ responses that are mainly represented by the *Peak* class and partly by the *Oscillation* & *Peak* class. The pronounced reduction in the *Oscillation* & *Peak* and *Peak* classes (**Figure 7C**) confirms this assumption. As shown in **Figure 7C**, the fraction of cells assigned to the *Peak* class is significantly reduced compared with controls. Interestingly, inhibiting PLC-pathways with U73122 and blocking P2X7 with minocycline did not affect SOCE in BV2 cells (**Figure 3D**). The former is consistent with our finding that probably, P2Y-activated, PLC-mediated pathways are not, or at least to a negligible degree, present in BV2 cells (**Figure 1B**), in contrast to primary microglia cells (Michaelis et al., 2015). Although not explicitly tested in primary microglia cells yet, one could extrapolate to strong blocking effect of U73122 on the P2Y-mediated transient Ca^2+^ release from internal stores measured in Ca^2+^-free solution (for example **Figure 2A**, Michaelis et al., 2015).

Wortmannin is a natural inhibitor of phosphatidylinositol 3-kinase and enhances microglia-mediated brain inflammation by promoting lipopolysaccharide (LPS)-induced NO release (Pyo et al., 2003). Besides a slightly increased fluorescence signal of cells treated with the drug (**Figure 8**), the data indicate no effect on ATP-induced Ca^2+^ signaling compared to controls. However, the presence of wortmannin resulted in reduced overall Ca^2+^ signals as compared to data with LY294 and AZ106 which is in line with a previous report on the suppressing effect of wortmannin on P2X4 in BV2 cells (Bernier et al., 2008).

LY294 is a morpholine-containing chemical compound and potent phosphoinositide 3-kinase inhibitor (Maira et al., 2009). The data in **Figure 8** suggest perturbation of ATP-induced Ca^2+^ signaling in cells displaying a sustained [Ca^2+^]_i_ increase only, but not in cells showing ‘*intermediate Ca^2+^ oscillations*’ plus sustained [Ca^2+^]_i_ increase.

AZ106 is a selective P2X7 antagonist and inhibits ATP-induced Ca^2+^ signaling (Bhaskaracharya et al., 2014). This is reflected by a strong decrease of cells with *Oscillation* & *Peak* and *Peak* classes, and an increase in the number of cells assigned to the *Other* class.

**FIGURE 9:**
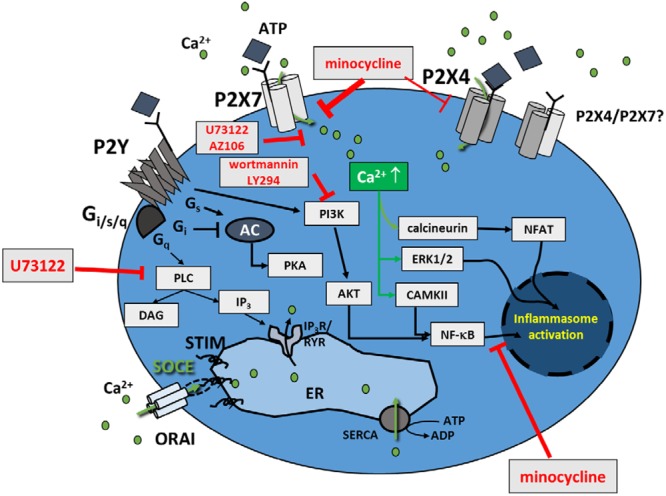
**Model of pathways involved in Ca^2+^ regulation in murine BV2 cells.** Apart from the purinergic ATP responses in BV microglia cells, either mediated through ionotropic P2X or metabotropic P2Y receptors, store-operated Ca^2+^ entry (SOCE) is also present in BV2 cells. Results from this study show that minocycline and U73122 do not affect SOCE, and SOCE does not affect low-ATP-induced P2X4-signaling. P2Y-signaling seems to be of only minor relevance in BV2 cells. Respective inhibitors of P2X/Y signaling used in the present study are indicated.

A model of the mode of actions of purinergic receptors (P2X, P2Y), and their involvement in downstream pro-inflammatory signaling and inhibitors used in this study is given in **Figure 9**.

In summary, our study shows the existence and crucial contribution of SOCE to ROCE in immortalized murine BV2 microglia. Interestingly, ATP appears to more selectively activate P2X than P2Y receptor responses in BV2 cells. Our ‘low-resolution’ high-content platform provides a more robust representation of purinergic Ca^2+^ signaling to overcome constraints of cell-to-cell variability in single cell high-resolution experiments. It will be useful for identifying compounds modulating microglial activation in the context of neuro-inflammation. To further develop our high-content screening platform to understand mechanisms of different purinergic phenotypes, future studies will include expression systems with defined receptor expression profiles, BV2 cells and primary cells, for comparison.

Although there are numerous examples of cell signaling studies carried out on BV2 cells alone, it is important to mention that some data obtained with cell lines may be prone to artifacts due to the immortalization procedure and may differ from data observed in primary cells. Thus, data reported in this paper are confined to BV2 cell and transferability has to be considered preliminary until cross-validation in primary microglia cells.

## Author Contributions

DA, OF, MS, LS, and DG designed research. OF, DG, RM, EZ, MS, KK, and AB performed research. OF, DG, RM, and AB analyzed data. OF, DG, and DA wrote the paper. All authors agreed on the final version of the manuscript.

## Conflict of Interest Statement

The authors declare that the research was conducted in the absence of any commercial or financial relationships that could be construed as a potential conflict of interest.
